# Antimicrobial resistance determinants in silage

**DOI:** 10.1038/s41598-022-09296-5

**Published:** 2022-03-28

**Authors:** Sára Ágnes Nagy, Adrienn Gréta Tóth, Márton Papp, Selçuk Kaplan, Norbert Solymosi

**Affiliations:** 1 Centre for Bioinformatics, University of Veterinary Medicine Budapest, 1078 Budapest, Hungary; 2 Department of Agricultural Biotechnology, Tekirdag Namik Kemal University, 59030 Tekirdag, Turkey

**Keywords:** Metagenomics, Antimicrobial resistance

## Abstract

Animal products may play a role in developing and spreading antimicrobial resistance in several ways. On the one hand, residues of antibiotics not adequately used in animal farming can enter the human body via food. However, resistant bacteria may also be present in animal products, which can transfer the antimicrobial resistance genes (ARG) to the bacteria in the consumer’s body by horizontal gene transfer. As previous studies have shown that fermented foods have a meaningful ARG content, it is indicated that such genes may also be present in silage used as mass feed in the cattle sector. In our study, we aspired to answer what ARGs occur in silage and what mobility characteristics they have? For this purpose, we have analyzed bioinformatically 52 freely available deep sequenced silage samples from shotgun metagenome next-generation sequencing. A total of 16 perfect matched ARGs occurred 54 times in the samples. More than half of these ARGs are mobile because they can be linked to integrative mobile genetic elements, prophages or plasmids. Our results point to a neglected but substantial ARG source in the food chain.

## Introduction

In intensive cattle farming, silage is an essential component of feed. An average dairy cow consumes 25–27 kg of this forage a day, reaching up to a silage consumption of 12,500 kg per lactation^1,2^. Silage is most commonly produced from maize or legume plants by the process of anaerobic fermentation. Throughout the fermentation process, fermenting microorganisms, including bacteria, multiply. As a result of this biochemical transformation, the silage is enriched with beneficial nutrients. If bacteria that are involved in the process harbor antimicrobial resistance genes (ARGs), the amount of these genes in the silage will increase in parallel with the bacterial counts. Consequently, silage, as a mass feed may continuously supply the gastrointestinal tract of animals with bacteria carrying ARGs. Bacteria entering the digestive system may come into contact with the host microbiota that facilitates the exchange of bacterial genes (e.g. ARGs) by horizontal gene transfer (HGT). HGT may take place as a result of three different processes: conjugation, transduction and transformation. Except for transformation, by which a bacterium can take up any gene from its environment, the routes of HGT require particular active delivery processes. By conjugation, cell-to-cell contact provides the opportunity for a copy of a plasmid to translocate to a recipient bacterium^3^. Transduction negates the condition of cell-to-cell contact, as in this case, bacteriophages act as a conduit for shuttling genes among bacteria^4^. The genetic environment of the genes involved in the transfer significantly influences the efficacy of the latter two HGT processes, i.e., the genes’ mobility. The reason why the mobility characteristics of ARGs involved in silage are worth taking into consideration is the following. If ARGs from silage are transmitted to pathogenic bacteria within an animal’s body, efficacy of antibiotic (AB) treatment may be reduced on the consequent bacterial diseases. In addition, in case of the gut colonization of silage-borne bacteria that carry ARGs, the appearance and enrichment of bacterial ARGs may take place in the animals’ environment after defecation. Decreased efficacy of AB treatments may result in economic loss, and the increased environmental ARG level may have additional veterinary and human health consequences. It is proven in former publications that the number of ARGs in fermented dairy products can increase due to the multiplication of fermenting bacteria^5^. Nevertheless, the description of this phenomenon cannot be found for silage in the literature. Our study aimed to examine the diversity, bacterial relatedness and mobility potential of ARGs deriving from silage. For this purpose, freely available next-generation sequencing (NGS) shotgun metagenome datasets were analyzed by a unified bioinformatics pipeline.

## Results

Based on the taxon classification performed on a database containing complete reference genomes of plants, the most dominant plants in the silage belong to the *Medicago* genus and most likely to the alfalfa (*M. sativa*) species. Further results of the analysis of the 52 shotgun metagenomic sequenced samples (Table 2) are summarised in the following sections. After presenting the bacteriome and the identified AGRs (resistome), predictions regarding the mobility potential of ARGs were also resumed based on genetic characteristics that may play a significant role in HGT.

### Bacteriome

**Figure 1:**
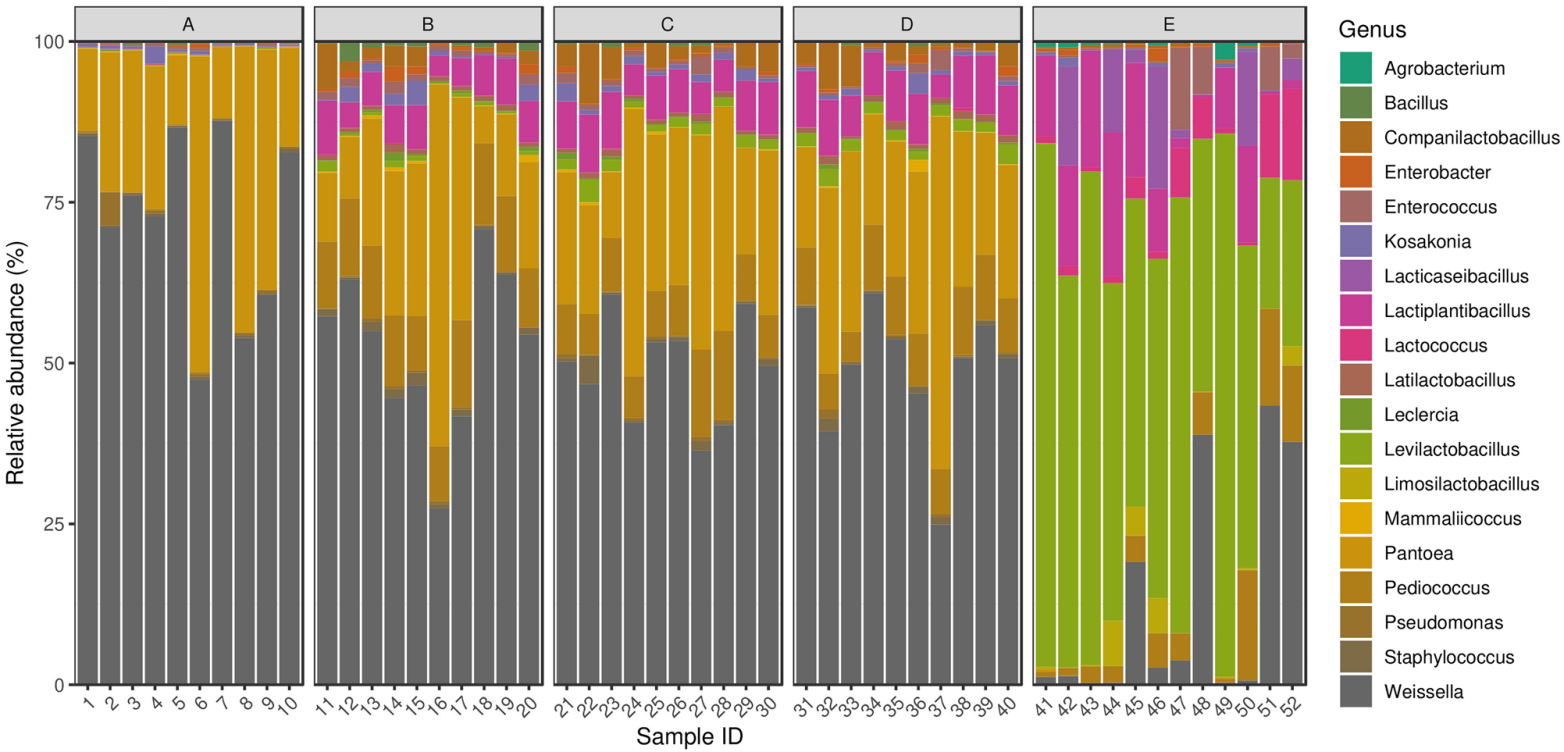
Silage core bacteriome. Relative abundances of genera that achieved more than 1% of the bacterial hits in any of the samples. The elements of the PRJNA495415 dataset were taken on days 0, 7, 14 and 28 were classified into groups A, B, C and D, respectively. All items from BioProject PRJNA764355 are assigned to group E.

By taxon classification, the number of reads aligning to bacterial genomes varied by samples (median: 20.6 × 10^6^, IQR: 2.9 × 10^6^). The relative abundances of genera that achieved more than 1% of the bacterial hits in any of the samples are shown in Fig. 1.

The dominant bacterial genera (with mean abundance) in descending order were *Weissella* (45.7%), *Pantoea* (18.5%), *Levilactobacillus* (13.5%), *Pediococcus* (6.7%), *Lactiplantibacillus* (6.3%), *Companilactobacillus* (1.7%), *Lacticaseibacillus* (1.3%), *Enterococcus* (1.2%), *Lactococcus* (1%), *Kosakonia* (0.8%), *Staphylococcus* (0.6%), *Enterobacter* (0.5%), *Latilactobacillus* (0.5%), *Bacillus* (0.4%), *Limosilactobacillus* (0.4%), *Pseudomonas* (0.4%), *Leclercia* (0.2%), *Mammaliicoccus* (0.2%), *Agrobacterium* (0.1%).

### Resistome

**Figure 2:**
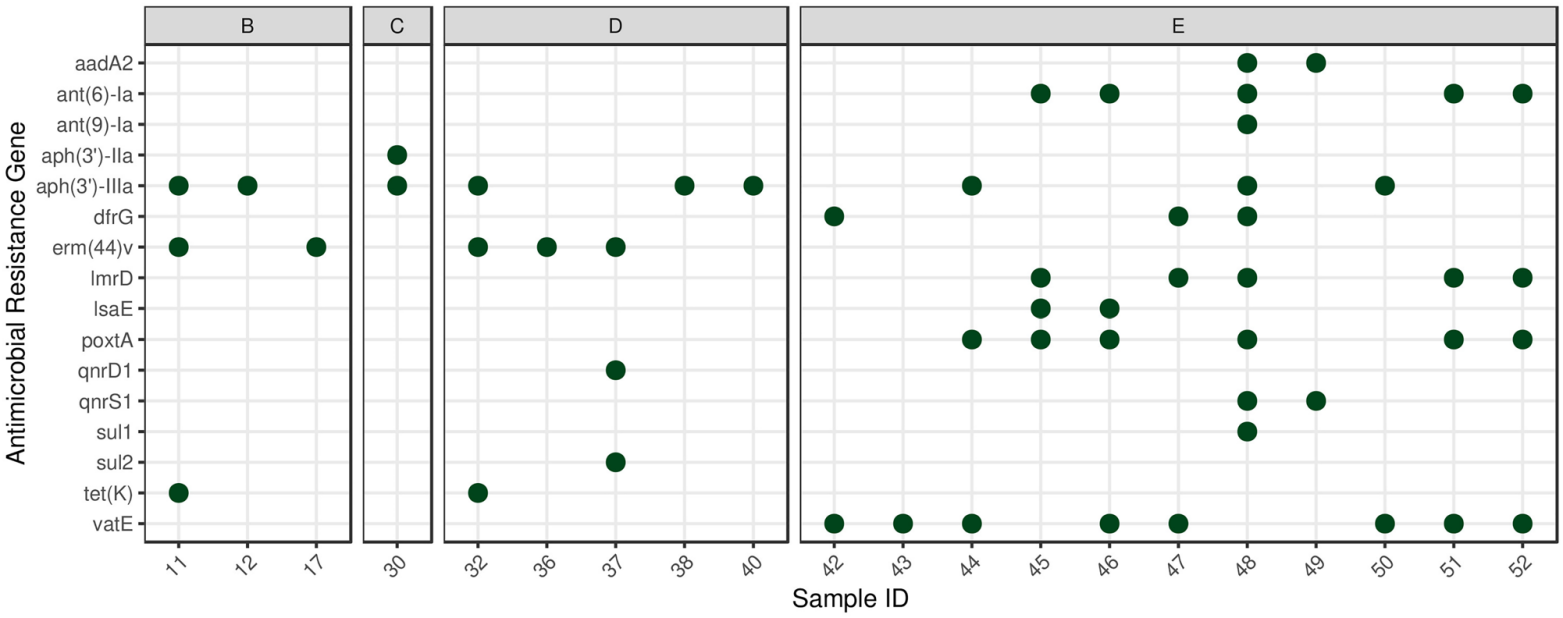
Identifed antimicrobial resistance genes (ARGs) by samples. Perfect ARG matches were plotted by samples. The data of the PRJNA495415 taken on days 0, 7, 14 and 28 were classified in groups A, B, C and D, respectively. All samples from BioProject PRJNA764355 were assigned to group E.

The median length of the filtered contigs harboring ARGs constructed by de novo assembly was 4,204 bp (IQR: 2,832). The number of ARGs found on the contigs ranged from 1 to 2. The identified 16 ARG types appeared 54 times in 20 of the analyzed 52 samples. These ARGs were the following: *aadA2*, *ant(6)-Ia*, *ant(9)-Ia*, *aph(3’)-IIa*, *aph(3’)-IIIa*, *dfrG*, *erm(44)v*, *lmrD*, *lsaE*, *poxtA*, *qnrD1*, *qnrS1*, *sul1*, *sul2*, *tet(K)*, *vatE* (Fig. 2). The resistance mechanism of identified ARGs was the antibiotic inactivation (48.1%), antibiotic target protection (20.3%), antibiotic efflux (13.0%), antibiotic target alteration (9.3%), antibiotic target replacement (9.3%) in descending order of frequency. Table 1 shows the bacterial species to which the ARG harboring contigs were assigned based by the taxon classification. In addition, the table also presents which drug classes are affected by the ARGs.

**Table 1:** Identified ARGs and the drug classes affected by them per bacterial species of origin.

Bacteria	ARG(s)	Drug class
Acinetobacter baumannii	aadA2	Aminoglycoside
Amylolactobacillus amylophilus	ant(6)-Ia	Aminoglycoside
Bacillus subtilis	aph(3’)-IIa	Aminoglycoside
Cronobacter sp. JZ38	qnrS1	Fluoroquinolone
Enterobacter hormaechei	aadA2, sul1	Aminoglycoside, sulfonamide
Enterococcus faecalis	aph(3’)-IIIa	Aminoglycoside
Enterococcus faecium	poxtA	Lincosamide, macrolide, oxazolidinone, phenicol, pleuromutilin, streptogramin, tetracycline
Escherichia coli	sul2	Sulfonamide
Gracilibacillus sp. SCU50	dfrG	Diaminopyrimidine
Lacticaseibacillus manihotivorans	ant(6)-Ia	Aminoglycoside
Lacticaseibacillus paracasei	poxtA	Lincosamide, macrolide, oxazolidinone, phenicol, pleuromutilin, streptogramin, tetracycline
Lactiplantibacillus plantarum	aph(3’)-IIIa, poxtA, vatE	Aminoglycoside, lincosamide, macrolide, oxazolidinone, phenicol, pleuromutilin, streptogramin, tetracycline
Lactococcus lactis	lmrD	Lincosamide
Levilactobacillus brevis	poxtA	Lincosamide, macrolide, oxazolidinone, phenicol, pleuromutilin, streptogramin, tetracycline
Ligilactobacillus acidipiscis	ant(9)-Ia	Aminoglycoside
Providencia rettgeri	qnrD1	Fluoroquinolone
Staphylococcus aureus	ant(6)-Ia, tet(K)	Aminoglycoside, tetracycline
Staphylococcus carnosus	erm(44)v	Lincosamide, macrolide, streptogramin
Staphylococcus pseudoxylosus	erm(44)v	Lincosamide, macrolide, streptogramin
Staphylococcus saprophyticus	erm(44)v	Lincosamide, macrolide, streptogramin
Streptococcus suis	lsaE	Lincosamide, macrolide, oxazolidinone, phenicol, pleuromutilin, streptogramin, tetracycline
Tetragenococcus halophilus	aph(3’)-IIIa	Aminoglycoside
Weissella paramesenteroides	ant(6)-Ia	Aminoglycoside

### Mobilome

**Figure 3:**
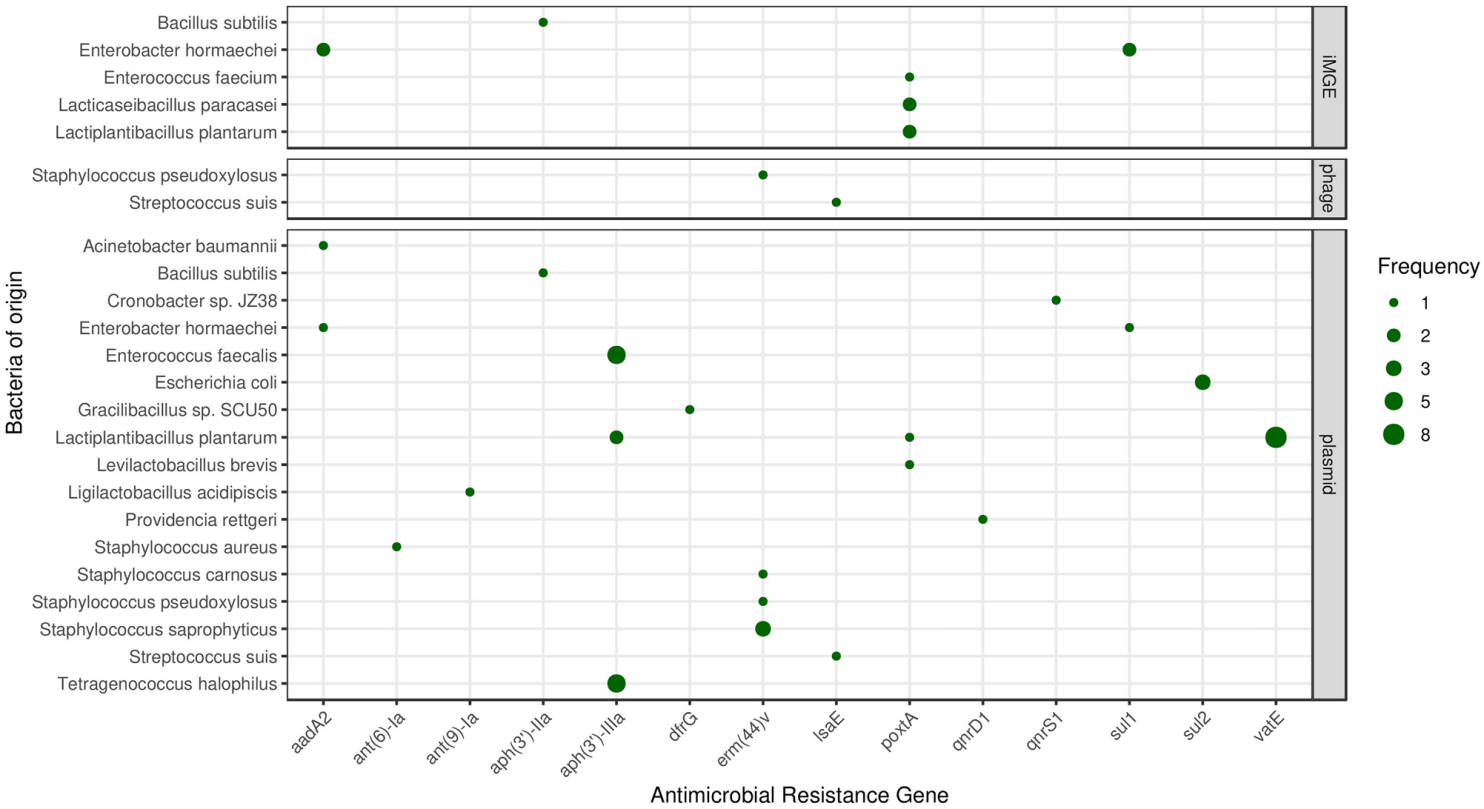
Mobile antimicrobial resistance gene frequency by bacteria of origin. The size of the dots indicates the occurrence frequency of the ARGs flanked by iMGE, positioned in a plasmid or a phage.

We found a total of 53 ARGs that are assumably mobile. Ten of these ARGs are linked to integrative mobile genetic elements (iMGE). A further two ARGs were detected in prophages and forty-one on plasmids. The frequencies of ARGs associated with iMGEs, phages and plasmids are summarized in Fig. 3 by bacterial species of origin.

Following the distance method proposed by Johansson et al.^6^, integrated mobile genetic element associated ARGs were detected in five samples (30, 45, 46, 48, 52) and five species (*B. subtilis*, *E. hormaechei*, *E. faecium*, *L. paracasei*, *L. plantarum*). *B. subtilis* associated *aph(3ʹ)-IIa* in sample 30, and *poxtA* of *E. faecium* in sample 45, of *L. plantarum* in sample 52, and of *L. paracasei* in sample 46 were detected as iMGE linked gene. *Sul1* and *aadA2* were detected in *E. hormaechei* co-existed with integrated mobile elements in sample 48. Two prophage-linked ARGs were identified, the contig harboring *erm(44)v* classified to *S. pseudoxylosus* by VirSorter2 was found to be of dsDNA phage origin while the contig of *lsaE* from *S. suis* was predicted as ssDNA derived. These phage associated ARGs were detected in sample 37 and sample 45 respectively. Contings with ARGs were predicted to belong to plasmids in 19 samples (Nr. 11, 12, 17, 30, 32, 36, 37, 40, 42, 43, 44, 45, 46, 47, 48, 49, 50, 51, 52).

## Discussion

Throughout our study, numerous perfect ARG matches were identified in the metagenome of *Medicago* silage samples. All but group A of the analyzed subsets had at least one sample containing one or more ARG. Among the PRJNA495415 Bioproject samples, the highest number of ARGs were found in group D. The interpretation of this finding is limited due to the lack of detailed information on the samples. Interestingly, all but one of the PRJNA764355 bioproject samples contained ARGs. Due to the lack of metadata, it is hard to find any reason for this high ARG level. However, one possible cause might be that the PRJNA764355 samples were sequenced deeper and thus contained approximately 1.3 times more reads than the PRJNA495415 samples. It is known from previous studies that deeper sequencing leads to the generation of more complete genes by the de novo assembly^5,7^.

In the following, our results will be interpreted from a perspective of bacteriological significance, genomic relevance and furthermore, antimicrobial stewardship and possible clinical aspects.

Taking the microbiome into consideration, bacteria that were predicted to harbor the identified ARGs can be classified according to their presence in silage. In the literature, the following bacteria are mentioned to be characteristic for silage: *B. subtilis*^8^ *E. faecium*^9^ *E. coli*^10^, *L. plantarum*^11^, *L. lactis*^12^, *L. brevis*^13–15^, *L. acidipiscis*^12^ *W. paramesenteroides*^16^. The genera of these species dominate the bacteriome of the samples. The identified *Cronobacter* sp. JZ38^17^ may be of plant origin. However, it can be assumed that other species may be present as contaminants of the silage: *A. amylophilus*, *E. hormaechei*, *E. faecalis*, *Gracilibacillus* sp. SCU50, *L. manihotivorans*, *L. paracasei*, *P. rettgeri*, *S. aureus*, *S. carnosus*, *S. pseudoxylosus*, *S. saprophyticus*, *S. suis*, *T. halophilus*. Nevertheless, some of these bacteria are members of the *Lactobacillaceae* family, the *Leuconostoc* or *Enterobacter* genera. Numerous species of these groups are typical for fermented food and feed components.

From a genomic point of view, the following was found in the literature regarding the co-occurrence of the ARGs identified in our study and the bacteria carrying them. *AadA2* encoding an aminoglycoside nucleotidyltransferase has been described in *A. baumanni* in former publications^18,19^. *ant(6)-Ia*, that is an aminoglycoside nucleotidyltransferase gene, appears in many species, including *Lactobacillus* spp.^20^. Its species-specific association with *A. amylophilus* has not been described in any former publications. *aph(3’)-IIa*, an aminoglycoside phosphotransferase^21^, to our knowledge, has not been detected in *B. subtilis* up untill now. *QnrS1* encoding a quinolone resistance protein was originally identified in *Shigella flexneri*^22^. In line with our results, this gene has recently been mentioned to appear in *Cronobacter* spp. in a case report^23^. *E. hormaechei* deriving *aadA2* and *sul1*, a sulfonamide resistant dihydropteroate synthase gene that is described to appear in Gram-negative bacteria^21^ have been reported to appear in the genom of *Enterobacter* spp. and *E. hormaechei*, respectively in former publications as well^24,25^. Within the *Enterococcus* genus, two perfect ARG matches were identified, namely *aph(3’)-IIIa* in *E. faecalis* and *poxtA* in *E. faecium*. *aph(3’)-IIIa* is an aminoglycoside phosphotransferase that normally appears in *S. aureus*^21^ and *Enterococcus* spp.^26^, while *poxtA* is a gene encoding an ABC-F subfamily (ATP-binding cassette-F) protein that facilitates resistance to tetracycline, phenicol, and oxazolidinone via modification of the bacterial ribosome. First detection of *poxtA* took place in a methicillin-resistant *S. aureus* strain^21^, followed by other bacterial species, including *E. faecium*^27^. *Sul2*, a sulfonamide resistant dihydropteroate synthase of Gram-negative bacteria is commonly described in *E. coli*^21,28^. *DfrG* is a plasmid-encoded dihydrofolate reductase^21^ that, to our knowledge, has not been described in *Gracibacillus* spp. up untill now, but has already appeared in the *Bacillaceae* family^29^. *ant(6)-Ia*, an aminoglycoside nucleotidyltransferase gene appears in many species, including *Lactobacillus* spp.^20^. Its species-specific association with *L. manihotivorans* has not been described in any publications. *PoxtA* that was detected in *L. paracasei*, *L. plantarum* and *L. brevis* in the silage samples, has been described to appear in *Lactobacillaceae*, namely *L. acidophilus*, but not in these very species^30^. Another species that was detected harboring *aph(3’)-IIIa* in the silage samples was *L. plantarum*. This finding is in line with the ARG-species match results mentioned in former publications^31^. Furthermore, *L. plantarum* was also associated with *vatE* that encodes an acetyltransferase conferring resistance against streptogramins^21^. *VatE* was originally found in *E. faecium*^21^ and has since then been identified in *Lactobacillaceae*^32^, but not specifically in *L. plantarum*. *L. acidipiscis ant(9)-Ia*, an aminoglycoside nucleotidyltransferase gene^21^ was associated with this genus for first within this study. Gene *qnrD1* encoding a quinolone resistance protein that is normally detected in *Salmonella enterica*^21^, has already been found in *Providencia* spp.^33^ and was attached to *P. rettgeri* in our study as well. *S. aureus* could have been associated with two ARGs, *ant(6)-Ia* and *tetK* encoding a tetracycline efflux protein, that are both common findings in *Staphylococcus* spp.^34,35^. Although, *erm(44)v* was first detected in the *S. saprophyticus*^36^, no literature could be found about the appearance of this gene in *S. carnosus* or in *S. pseudoxylosus* species. *lsaE* encoding another ABC-F subfamily protein conferring resistance to pleuromutilin, lincosamide, and streptogramin A is a common finding in *Streptococcus* spp.^37^ and has also been associated with *S. suis* in previous publications^38^. Besides the bacterial species mentioned above, *aph(3’)-IIIa* was also detected in *T. halophilus*. This ARG is often appears in *Enterococcaceae*^21^ but has not yet been written down in this species. Furthermore, to our knowledge, *W. paramesenteroides* associated *ant(6)-Ia* has first been detected in this study.

Throughout our study, several ARGs were predicted to be co-occurring with genetic attributes facilitating mobility. The bioinformatic analysis of the mobility characteristics relied upon the identification of three major mobility determination groups, namely iMGEs, phages and plasmids. We found *aph(3’)-IIa* linked to an integrated mobile genetic element in *B. subtilis* that is in line with similar findings of *E. coli*.^21^ While *aadA2* and *sul1* have both been described to appear on plasmids in *E. hormaechei*^39^, we found them associated with iMGEs. Our finding on iMGE flanked *poxtA* in *E. faecium* is in line with the current literature^40^. We found the same co-occurrence, namely *poxtA* and an iMGE, in *L. paracasei*. This phenomenon has not been published in that species to the best of our knowledge. Gene *erm(44)v* and *lsaE* were associated with prophages in *S. pseudoxylosus* and *S. suis*. While a similar linkage can be found in the literature in connection with *erm(44)v*^41^, no details of mobility characteristics are mentioned in a recent report of the latter gene^38^. All other mobile ARGs were detected on contigs that were predicted to derive from plasmids. In case of *aadA2* in *A. baumannii*^21^; *aadA2* and *sul1* in *E. hormaechei*^21^; *aph(3’)-IIIa* in *E. faecalis*^21^; *sul2* in *E. coli*^21^; *aph(3’)-IIIa* in *L. plantarum*^42^; *qnrD1* in *P. rettgeri*^21^; *ant(6)-Ia* in *S. aureus*^21^ and *erm(44)v* in *S. saprophyticus*^41^ plasmid associations have been formerly described in the literature. To our knowledge, no publications have yet been released on the plasmid occurence of *aph(3’)-IIa* in *B. subtilis*, *dfrG* in *Gracilibacillus* sp. SCU50, *ant(9)-Ia* in *L. acidipiscis*, *lsaE* in *S. suis*, *qnrS1* in *Cronobacter* sp. JZ38, *erm(44)v* in *S. carnosus* and *S. pseudoxylosus*. Hao et al. described *poxtA* embedded in a multi-resistance plasmid with mobile elements flanking in *E. fecalis*. This gene has been found in a number of Gram-positive bacteria, including enterococci as well, but it has neither been identified in *L. plantarum* nor *L. brevis*^43^. Previous findings confirm the occurrence of *vat(E)* on plasmids^44^. Nevertheless, in spite of its frequent presence in enterococci^45^ there is no evidence of its former plasmid-associated appearance in *L. plantarum*. We found that gene *aph(3’)-IIIa* of *T. halophilus* was encoded on a plasmid that is consistent with the fact that *aph(3’)IIIa* is often identified on high molecular weight plasmids and chromosomes of the enterococcal species^46^. Nonetheless, to the best of our knowledge, a description of the *aph(3’)-IIIa* gene in *T. halophilus* is a pioneer finding.

The mobility characteristics of the ARGs may not only provide us with information regarding the public health risk that may be associated with the samples, but also point to the possible origins of the genes. Regardless of human intervention, ARGs are present in the microbial communities^47^. However, antimicrobial use and abuse intensifies the horizontal transfer of ARGs and thus contributes to the spread of AMR. In the animal production sector, the use of antibiotics is common, thus bacteria appearing in the feces and in the surroundings of the animals (e.g. in farm air, on tools, vehicles or other settings related to animals) often harbor bacteria with an advanced ARG set. Silage may get in direct physical contact with these bacteria at the farms and thus get contaminated with a few ARGs. Consequently, the presence of ARGs in the silage samples was well-expected, but the abundance of resistance genes and MGEs may increase due to the application of antibiotics.

Examining further aspects of antimicrobial stewardship and possible clinical relevance, phenotypical manifestations and public health considerations associated with the detected ARGs are both important. Intense antimicrobial use (AMU) can be associated with the headway of AMR, as antibiotic pressure selects for bacteria carrying ARGs that facilitate bacterial survival. Quantifically, the majority of AMU around the globe occurs in agricultural settings^48,49^. Intensive farming, that serves to fulfill the high global demand for animal proteins relies on an antibiotic infrastructure to treat and prevent disease and occasionally, to increase feed efficacy. In order to maximize economic gains, few countries still apply regulations that facilitate the use of low doses of antibiotics as growth promoters^50^, while other regions, like the U.S. or Europe, have banned this practice. Nevertheless, besides the treatment of symptomatic infectious diseases, antibiotics are still widely used in the livestock sector for metaphylactic and prophylactic purposes in higher doses^51,52^. Even though, compared to the poultry and pig production sector, average antibiotic usage has relatively lower rates by cattle^53^, antimicrobial compounds are often chosen in this species as well. In cattle farming, mastitis is the most predominant reason for the administration of antibiotics by adult cattle, while enteritis and pneumonia is the most common reason for calves^54,55^. According to various reports and studies from around the world^54,56,57^ tetracyclines are of inevitable significance in the medication of cattle, while beta-lactams, macrolides, sulfonamides, lincosamides and ionophore antibiotics are also very widely used. Of the European Medicines Agency (EMA) Highest-Priority Critically Important Antibiotics (HPCIAs), namely third and fourth generation cephalosoprines, fluoroquinolones and polymixins, polymixins and fluoroquinolones are the most applied, although their sales rates are still far below the most frequently administered antibiotic groups by livestock species^57^. In our samples *E. faecium*, *L. paracasei*, *L. plantarum*, *L. brevis*, *S. aureus* and *S. suis* harbored genes, namely *poxtA* and *lsaE* that may confer resistance against multiple antibiotic groups, including tetracyclines. Moreover, *poxtA* was detected in the proximity of iMGEs in *L. paracasei* and *E. faecium* and harbored on a plasmid in *L. brevis*. In line with our findings, *Enterococcus* species related to cattle were heavily associated with tetracycline resistance by other authors too^58^. At some species *poxtA* and *lsaE* were even predicted to co-occur with more than one MGE type. In the genome of *L. plantarum poxtA* was predicted to be positioned on a plasmid and associated with an iMGE, while *S. suis* associated *lsaE* was located on a plasmid attached to a phage. Such genetic features may contribute to the horizontal transfer of ARGs among bacteria which is of outstanding clinical relevance in the case of such a commonly applied antibiotic group in cattle medicine, as tetracyclines. Perfect matches of genes conferring resistance against other clinically significant antibiotic groups, such as macrolides and sulfonamides were also identified in the genome of *E. hormaechei*, *E. faecium*, *E. coli*, *L. paracasei*, *L. plantarum*, *L. lactis*, *L. brevis*, *S. carnosus*, *S. pseudoxylosus*, *S. saprophyticus* and *S. suis*. Of these genes, *lsaE*, *sul1*, *sul2* and *poxtA* were even predicted to have enhanced mobility due to their association with multiple MGE groups. The only perfect match for an ARG against HPCIAs, *qnrS1*, that can confirm resistance against fluoroquinolones, has been detected in a *Cronobacter* spp. The presence of several ARGs presumably associated with iMGEs in the feed of dairy cows harbors the potential to affect the resident microbiota of the animals. As *B. subtilis* and *E. faecium* frequently appear in probiotics for cattle^59,60^ it is possible that some microorganisms colonize niches in the foregut and proliferate the ARGs they possess. However, even if they cannot reproduce in the ruminal environment, ARGs can still be disseminated through horizontal gene transfer, especially in the presence of antibiotic therapy. Furthermore, ARGs can possibly spread further, to lower gastrointestinal (GI) regions. Fecal microbiota transfer administered to the stomach could restore the microbial population of the colon in human patients^61^, indicating a high volume of viable bacteria reaching the distal regions. Similar results were found in cattle with rumen microbiota transplantation affecting the microbial population of the hindgut^62^. If ARGs spread all around the GI tract, serious animal and public health concerns could be raised. Among enteral diseases, salmonellosis is the major indication of antibiotic therapy in dairy cattle. Enhanced antibiotic resistance of these bacteria could contribute to the economic loss from the disease as many strains already exhibit resistance to several antibiotics^63^. Furthermore, during pathological conditions, like ruminal acidosis, bacteria can translocate to distant locations in the host’s body. Interestingly bacterial translocation was even described in the absence of GI diseases in case of specific microorganisms in humans and rodents^64,65^.

As a consequence of the colonization and possible ARG proliferation processes, pathologies caused by phenotypically resistant bacteria can induce animal welfare and economic issues. Animals harboring ARGs in their gut can contaminate their environment with ARGs through fecal matter as well as farm workers who get in direct contact with the animals, even consumers of dairy products can be affected, as farm animal-borne bacteria that harbor potentially mobile ARGs^66–68^ might be distributed by products intended for human consumption. For instance, we have previously found ARGs in raw milk samples provided for human consumption^69^. Fecal contamination during milking^70^ is a possible way of ARG transfer into raw milk, however other routes are also possible. In humans and in rodents for instance, maternal mononuclear cells transfer microorganisms to milk during lactation^66,67^. The possibility for this phenomenon was described in cattle as well^68^.

ARGs that are transferred to the human body through these routes might decrease the efficacy of antibiotic therapy. In order to gain a deeper insight into the exact role of silage in possible ARG transmission processes, many points still need to be examined and clarified. It would be essential to analyse the colonization success of ARG harboring silage-borne bacteria that enter the body of animals and the extent of ARG transfer of invasive donor bacteria to recipient bacteria living in the gastrointestinal tract. The silage involved in the study is of *Medicago* origin, and our results are based on data from only two projects. Hence, it would also be necessary to investigate the ARG content of other alfalfa and corn silages.

Antimicrobial resistance is an emerging global threat to public health that both affects agriculture and the healthcare sector. The usage of antibiotics in livestock species exceeds the rate of human applications^71^. Antibiotic use in food animal medicine is also considered a risk as it may provide an indirect transfer route of antibiotic residual^72^ ARGs via the food chain^73^. Even though antimicrobials administered for veterinary use, may exert an undesired effect on the food chain, the presence of ARGs in dairy cattle nutrition research is still underrepresented in the literature. According to our results, microbial mass contained in fermented feeds have other medical risks than transmitting contagious diseases, like listeriosis^74^. The bacterial content of these mass feeds, that is either, required for the fermentation processes or collected from various sources of contamination on the farms, could play an essential role in the ARG shift through the food chain.

## Materials and methods

### Data

We searched appropriate datasets in the National Center for Biotechnology Information (NCBI) Sequence Read Archive (SRA) repository. In December 2021, only two shotgun metagenomic BioProjects (PRJNA495415^75^, PRJNA764355) could have been found that had adequate depth for the de novo assembly that our study is based on. The median read count (interquartile range, IQR) of the samples was 26*.*5 × 10^6^ (3*.*0 × 10^6^) and 34*.*7 × 10^6^ (1*.*5 × 10^6^) in datasets PRJNA495415 and PRJNA764355, respectively. There is limited metadata available of the samples in the NCBI SRA database (Table 2). Nevertheless, it can be assumed from the metadata that the samples of PRJNA495415 were taken at different fermentation periods. Samples were taken on days 0, 7, 14 and 28 were classified in groups A, B, C and D, respectively. Based on metadata of PRJNA764355 samples, no such stratification was possible, so all samples were classified as group E.

**Table 2:** Analyzed samples.

BioProject				PRJNA495415				PRJNA764355	
Group	A		B		C		D		E
Id	Run	Id	Run	Id	Run	Id	Run	Id	Run
1	SRR7990583	11	SRR7990582	21	SRR7990580	31	SRR7990581	41	SRR16036389
2	SRR7990587	12	SRR7990586	22	SRR7990585	32	SRR7990584	42	SRR16036390
3	SRR7990591	13	SRR7990590	23	SRR7990589	33	SRR7990588	43	SRR16036391
4	SRR7990592	14	SRR7990593	24	SRR7990594	34	SRR7990595	44	SRR16036392
5	SRR7990598	15	SRR7990599	25	SRR7990596	35	SRR7990597	45	SRR16036393
6	SRR7990604	16	SRR7990605	26	SRR7990600	36	SRR7990601	46	SRR16036394
7	SRR7990608	17	SRR7990609	27	SRR7990602	37	SRR7990603	47	SRR16036395
8	SRR7990610	18	SRR7990611	28	SRR7990606	38	SRR7990607	48	SRR16036396
9	SRR7990612	19	SRR7990613	29	SRR7990614	39	SRR7990615	49	SRR16036397
10	SRR7990616	20	SRR7990617	30	SRR7990618	40	SRR7990619	50	SRR16036398
								51	SRR16036399
								52	SRR16036400

The samples of dataset PRJNA495415 were taken on days 0, 7, 14 and 28 were classified in groups A, B, C and D, respectively. All samples from BioProject PRJNA764355 are assigned to group E. Column Run contains the National Center for Biotechnology Information (NCBI) Sequence Read Archive (SRA) run identifiers.

### Bioinformatic analysis

Quality based filtering and trimming of the raw short reads was performed with TrimGalore (v.0.6.6, https://github.com/FelixKrueger/TrimGalore), setting 20 as a quality threshold. Only reads longer than 50 bp were retained and taxonomically classified using Kraken2 (v2.1.1)^76^ and a database created (24/03/2021) from the NCBI RefSeq complete archaeal, bacterial, viral and plant genomes. For this taxon assignment the -confidence 0.5 parameter was used to obtain more precise species level hits. The taxon classification data was managed in R^77^ using functions of the packages phyloseq^78^ and microbiome^79^. The preprocessed reads were assembled to contigs with MEGAHIT (v1.2.9)^80^ using default settings. The contigs were also classified taxonomically with Kraken2 with the same database as above. From the contigs all possible open reading frames (ORFs) were gathered with Prodigal (v2.6.3)^81^. The protein translated ORFs were aligned to the ARG sequences of the Comprehensive Antibiotic Resistance Database (CARD, v.3.1.3)^21,82^ by Resistance Gene Identifier (RGI, v5.2.0) with Diamond^83^. ORFs having a perfect match against the CARD database were exclusively kept for further analysis. Integrative mobile genetic element (iMGE) content of contigs harboring ARG was analyzed with MobileElementFinder (v1.0.3) and its database (v1.0.2)^6^. Following the distance concept of Johansson et al.^6^ for each bacterial species, only those with a distance threshold defined within iMGEs and ARGs were considered associated. In the MobileElementFinder database (v1.0.2) for *E. hormaechei*, the longest composite transposon (cTn) was the *Tn3000*. In case of this species, its length (11,823 bp) was taken as the cut-off value. For *E. faecium*, this threshold was the length of the *Tn6246* transposon, namely 5,147 bp. As the database neither contains species-level, nor genus-level cTn data for *Bacillus*, *Lactiplantibacillus* and *Lacticaseibacillus* species, a general cut-off value was chosen for the contigs of these species. This value was declared as the median of the longest cTns per species in the database (10,098 bp). The plasmid origin probability of the contigs was estimated by PlasFlow (v.1.1)^84^ The phage content of the assembled contigs was prediced by VirSorter2 (v2.2.3)^85^. The findings were filtered for dsDNAphages and ssDNAs. All data management procedures, analyses and plots were performed in R environment (v4.1.0)^77^.

## Data Availability

The datasets analysed in the current study are available in the National Center for Biotechnology Information (NCBI) Sequence Read Archive (SRA) repository and can be accessed through the PRJNA495415 and PRJNA764355 BioProject identifiers.
